# Heterologous expression of xanthophyll esterase genes affects carotenoid accumulation in petunia corollas

**DOI:** 10.1038/s41598-020-58313-y

**Published:** 2020-01-28

**Authors:** Sanae Kishimoto, Chihiro Oda-Yamamizo, Akemi Ohmiya

**Affiliations:** 1 Institute of Vegetable and Floriculture Science, National Agriculture and Food Research Organization (NARO), Fujimoto 2-1, Tsukuba, Ibaraki, 305-0852 Japan; 2 Japanese Society for the Promotion of Science (JSPS), Tokyo, 102-0083 Japan

**Keywords:** Molecular engineering in plants, Plant sciences, Plant breeding

## Abstract

The majority of carotenoids in petals are xanthophylls and most of these xanthophylls are esterified with fatty acids. Although petunia (*Petunia x hybrida*) is an important ornamental plant, it cannot accumulate enough carotenoids to have deep-yellow flowers. Our previous study suggested that low esterification activity causes low carotenoid accumulation in petunia corollas. Here, we introduced *xanthophyll esterase* (*XES*) from the petals of *Ipomoea obscura*, tomato (*Solanum lycopersicum*), and marigold (*Tagetes erecta*) into a pale-yellow-flowered cultivar of petunia to see whether these affect carotenoid accumulation in petunia corollas. Carotenoid contents and the proportions of esterified xanthophylls were elevated in the corollas of *XES*-overexpressing (*XES*-OX) transformants. Expression analysis showed that the transcript levels of endogenous carotenoid biosynthetic genes, which included *geranylgeranyl diphosphate synthase* 2, *ζ-carotene desaturase*, and *lycopene β-ring cyclase* in corolla tubes were upregulated in *XES*-OX plants. In addition, we discovered a difference in the composition of esterified xanthophylls among *XES*-OX plants, which may be caused by differences in the substrate specificity of their respective XESs. We conclude that esterification is an important process for carotenoid accumulation and *XES* is a useful tool for the quantitative and qualitative control of carotenoid accumulation in petals.

## Introduction

Carotenoids are important pigments for petal coloration in the yellow to red range. In flowers, the main role of these pigments is to attract insects that help pollination. Carotenoid components in petals vary widely among plant species, but the majority are xanthophylls (oxygenated carotenoids)^1,2^. There is increasing evidence that carotenoid accumulation in petals is regulated predominantly at the transcriptional level of carotenoid biosynthesis and catabolic genes^3^. Asiatic hybrid lily^4^ and *Ipomoea* species^5^ show higher expression of carotenoid biosynthetic genes in carotenoid-rich yellow or orange petals than in white petals. On the other hand, in some plant species, carotenoid cleavage dioxygenase (CCD) has a significant impact on carotenoid accumulation in petals^6–11^. The best-known example is chrysanthemums: *CCD4a* is expressed only in white petals, and suppression of *CCD4a* expression results in an increased carotenoid accumulation in petals^10^. The results indicate that carotenoids are synthesized, but are subsequently degraded by CCD4a, which results in the white petal color in chrysanthemum. Higher level of carotenoids in pale yellow petals in *Eustoma* and petunia is attributed to the higher expressions of biosynthetic genes and lower expression of *CCD4* than in white petals^12,13^. Judging from the analysis of biosynthetic and catabolic gene expressions, amounts of carotenoids in petals might be mainly determined by the balance of biosynthesis and degradation.

Another important factor that affects carotenoid accumulation in petals is xanthophyll esterification. Most xanthophylls in petals are esterified with fatty acids via oxygenated residues^5,14–17^, and accumulate in chromoplasts, which differentiate from chloroplasts during floral development^18,19^. In carotenoid-rich petals, carotenoids in the chromoplast are found in carotenoid-lipoprotein sequestering substructures, which consist of carotenoids, polar lipids, and enzymatic and structural proteins. These substructures are classified into fibrillar, globular, and tubular types^20,21^. Deruère *et al*. reported that fibril assembly *in vitro* was more efficient with esterified xanthophylls than with free-form xanthophylls or carotenes^22^. They presumed that this sequestration mechanism enables the cells to accumulate massive amounts of carotenoids. Therefore, we consider that esterification may enhance xanthophyll accumulation and cause deeper yellow coloration in flowers. Ariizumi *et al*. identified a tomato (*Solanum lycopersicum* L.) mutant, pale yellow petal 1 (*pyp1*), which lacks esterified xanthophylls in flowers^17^. Complementation of the *PYP1* gene into the *pyp1* mutant recovered esterified xanthophylls to the level of wild-type (WT) plants, indicating that PYP1 functions as a xanthophyll esterase (XES). Furthermore, since the *pyp1* mutant showed abnormal chromoplast development, they presumed that accumulation of esterified xanthophylls is important for normal chromoplast development. They proposed that PYP1 might be a key enzyme responsible for the high carotenoid accumulation in petals of various plants, including tomato.

Petunia (*Petunia x hybrida*) is an important ornamental plant and an excellent model plant for investigating floral pigmentation^23^. Although there are no deep-yellow-flowered petunia cultivars, there are pale-yellow-flowered cultivars that accumulate a small amount of carotenoids in their corollas^24,25^. To determine why petunias are unable to accumulate enough carotenoids to produce deep-yellow flowers, we previously compared carotenoid profiles and expression of carotenoid metabolic genes between pale-yellow-flowered petunia and deep-yellow-flowered calibrachoa (*Calibrachoa x hybrida*), a close relative of petunia^26^. We showed that the carotenoid contents and proportions of esterified xanthophylls in the corollas of petunia were significantly lower than in calibrachoa. In addition, we assumed petunia XES to have low substrate specificity for *trans*-xanthophylls, which are more abundant than *cis*-xanthophylls in corollas. These results suggest that a major reason for low carotenoid accumulation in petunia corollas is low esterification activity.

Here, we made transgenic petunia plants overexpressing *XES* from a yellow-flowered variety of *Ipomoea obscura* (L.) Ker Gawl., tomato, and marigold (*Tagetes erecta* L.). Petals of these plants had large amounts of esterified *trans*-xanthophylls with various compositions, suggesting that the introduced XESs had high esterification activity in respect of *trans*-xanthophylls and different substrate specificities^5,15,17^. We demonstrated that overexpression of these *XES* genes promoted the esterification of xanthophylls, development of carotenoid-lipoprotein sequestering substructures in chromoplasts, and overexpression of three endogenous carotenoid biosynthetic genes, resulting in an increase in total carotenoid content in the petunia corollas. Additionally, we showed that substrate specificity of XES affected the carotenoid profiles in the corollas of the transgenic plants.

## Results

### Isolation of full-length cDNA of *XES* from *I. obscura* and marigold and production of *XES-*overexpressing petunia plants

We isolated full-length cDNA sequences of *XES* from petals of *I. obscura* (*IoXES*) and marigold (*TeXES*). The deduced amino acid sequences covered 721 aa (IoXES) and 709 aa (TeXES); that of tomato PYP1 (SlXES) showed 59% similarity with that of IoXES and 55% similarity with that of TeXES. Tomato and petunia belong to the same Solanaceous family and SlXES and PhXES showed high similarity in the phylogenetic tree. In addition, IoXES and TeXES belong to different families, but the similarity of these 4 XESs was relatively high. An XES ortholog of *Arabidopsis*, known to function as phytyl ester synthase 1, and these 4 XESs were classified into different clades (Fig. S1).

**Figure 1:**
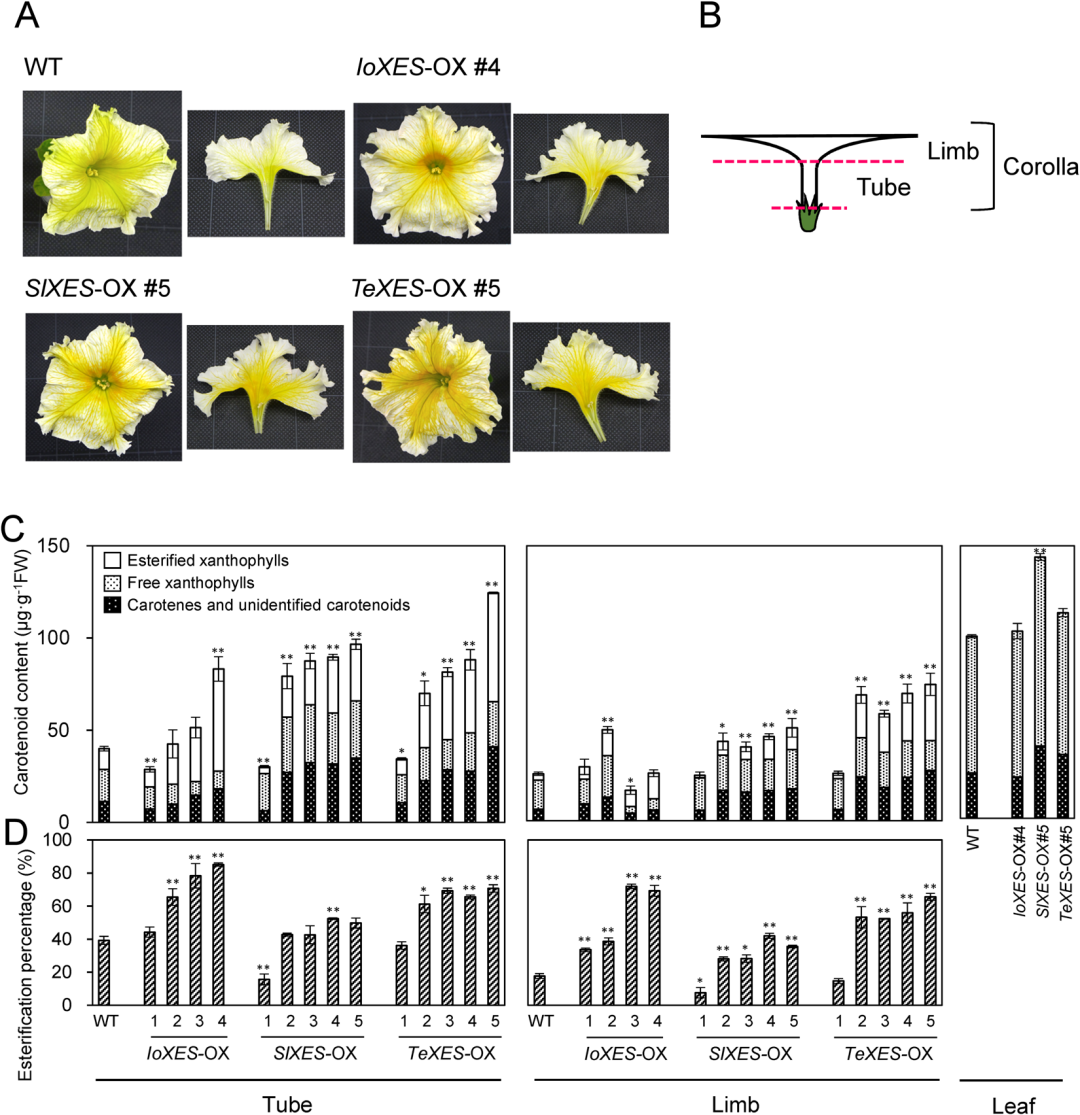
Phenotypes of *XES*-OX plants. (**A**) Flowers of *XES*-OX and WT plants on flowering day. (**B**) Sampling positions. (**C**) Total carotenoid content. Data obtained from leaves of *IoXES-*OX #4, *SlXES*-OX #5, and *IoXES*-OX #5 were shown as representatives of each line. (**D**) Percentage of esterified xanthophylls to total xanthophylls in corolla tubes, corolla limbs, and leaves of *XES*-OX and WT plants. Analyses were performed in triplicate. Means ± SE are shown. *P < 0.05, **P < 0.01 versus WT (Student’s *t-*test).

We then introduced constructs overexpressing *IoXES*, *SlXES*, or *TeXES* into petunia ‘California Girl’, which has pale yellow flowers, and obtained 6, 10, and 8 independent lines of transformants (OX), respectively. We chose four *IoXES*-OXs, five *SlXES*-OXs, and five *TeXES*-OXs for further analyses. Each group consisted of one transformant whose corolla color was unchanged (#1 transformant in all lines) and others whose corollas were deeper yellow than the WT plants (Fig. 1A).

### Comparison of carotenoid profiles between *XES*-OX and WT plants

**Figure 2:**
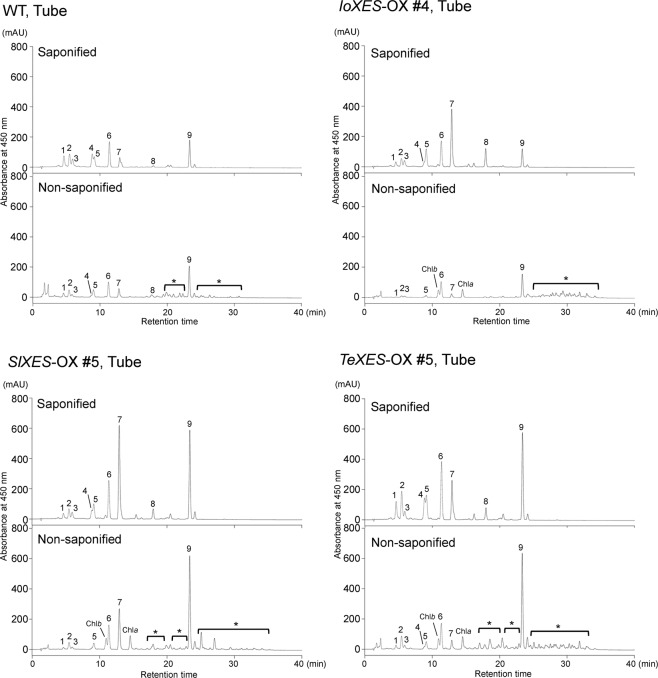
HPLC chromatograms of carotenoid extracts from corolla tubes of *XES*-OX and WT plants. Saponified and non-saponified carotenoid extracts from 0.025 g fresh weight of tubes were analyzed by HPLC. Peaks: (1) (all-*E*)-neoxanthin; (2) (all-*E*)-violaxanthin; (3) (9′*Z*)-neoxanthin; (4) (9*Z*)-violaxanthin; (5) unknown xanthophyll; (6) (all-*E*)-lutein; (7) (all-*E*)-antheraxanthin + (all-*E*)-zeaxanthin; (8) (all-*E*)-β-cryptoxanthin; (9) (all-*E*)-β-carotene; Chl*a*, chlorophyll *a*; Chl*b*, chlorophyll *b*. Asterisks shows peaks of esterified xanthophylls. Y-axis indicates milli-absorbance units (mAU).

In corolla tubes and limbs (Fig. 1B) and leaves, we compared carotenoid content between the WT and *XES*-OX plants (Figs. 1C,D and 2). All transgenic plants had significantly higher total carotenoid content than WT plants except for the tubes and limbs of the #1 plants (*IoXES*-OX #1, *SlXES*-OX #1, and *TeXES*-OX #1), the tubes of *IoXES*-OX #2 and #3, and the limbs of *IoXES*-OX #3 and #4 (Fig. 1C). *TeXES*-OX #5 had the highest carotenoid content in both tubes (3.11 × the WT value) and limbs (2.90×). Leaves of the *SlXES*-OX #5 accumulated significantly more carotenoids than those of *IoXES*-OX #4, *TeXES*-OX #5, and WT plants. HPLC analysis detected esterified xanthophylls in the non-saponified samples at a retention time of approximately 20 to 30 min in all limbs and tubes of *XES*-OX and WT plants tested (see examples in Fig. 2). The total amounts of esterified xanthophylls were significantly higher in all *XES*-OX plants except for #1 plants than in WT plants (Fig. 1C). Increase in esterification percentage was mostly associated with increase in total carotenoid content in the transgenic plants except in the limbs of *IoXES*-OX #3 and #4 (Fig. 1C,D). As observed for total carotenoid content, the tubes and limbs of the #1 plants showed a similar or lower esterification percentage than that of WT plants; the one exception was the limbs of *IoXES*-OX #1, which showed a significantly higher esterification percentage than WT. No esterified xanthophylls were detected in all leaves (Fig. 1C).

We tested the relationship between esterification percentage and total carotenoid content by Pearson’s correlation analysis (Fig. S3). There was a weak positive correlation between esterification percentage and total carotenoid content in all limb and tube samples (*r* = 0.396, *p* = 0.030; WT was included in all analyses). The trends differed between *IoXES*-OX and *SlXES*-OX/*TeXES* plants. In *SlXES*-OX and *TeXES*-OX plants, there was a strong positive correlation between esterification percentage and total carotenoid content (*SlXES*-OX: *r* = 0.829, *p* < 0.001; *TeXES*-OX: *r* = 0.911, *p* < 0.001). In *IoXES*-OX plants, there was a moderately positive correlation between esterification percentage and total carotenoid content in all samples (*r* = 0.459, *p* = 0.182).

**Figure 3:**
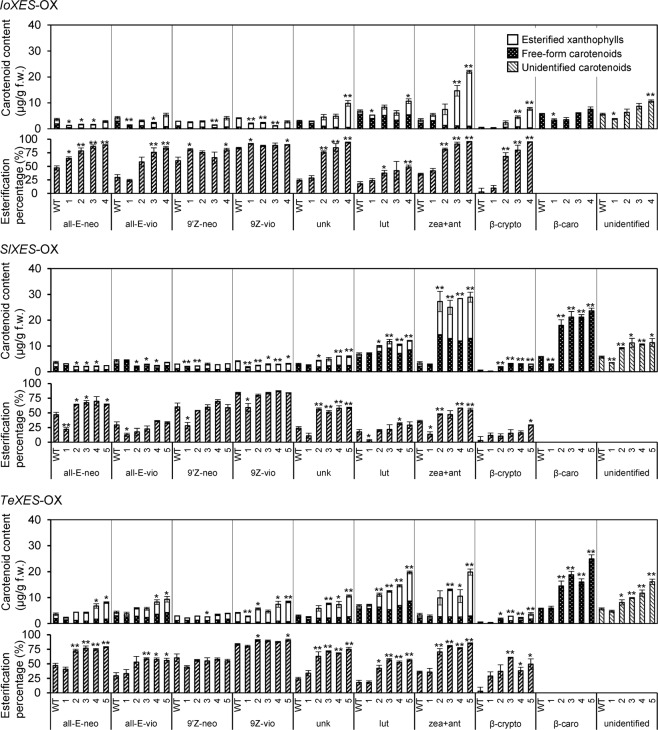
Content and esterification percentage of each carotenoid component in corolla tubes of *XES*-OX and WT plants. all-E-neo, (all-*E*)-neoxanthin; all-E-vio, (all-*E*)-violaxanthin; 9′Z-neo, (9′*Z*)-neoxanthin; 9Z-vio, (9*Z*)-violaxanthin; unk, unknown xanthophyll; lut, (all-*E*)-lutein; zea + ant, (all-*E*)-zeaxanthin + (all-*E*)-antheraxanthin; β-crypto, (all-*E*)-β-cryptoxanthin; β-caro, (all-*E*)-β-carotene; unidentified, unidentified carotenoids. Analyses were performed in biological triplicate. Means ± SE are shown. *P < 0.05, **P < 0.01 versus WT (Student’s *t*-test).

We detected esterified and free-form xanthophylls and β-carotene by HPLC (Fig. 2) and then compared the content and the esterification percentage of each carotenoid component between OX and WT plants (Fig. 3). The trends in content and esterification percentage of the various carotenoid components depended on the origin of the introduced *XES*, as outlined below.

In *IoXES*-OX plants #3 and #4, contents of zeaxanthin + antheraxanthin and β-cryptoxanthin were significantly higher than those in WT plants, whereas in all *IoXES*-OX plants, the contents of (all-*E*)-neoxanthin, (all-*E*)-violaxanthin, (9′*Z*)-neoxanthin, and (9*Z*)-violaxanthin were significantly lower than or not significantly different from those in WT plants. In all *IoXES*-OX plants except #1, percentages of xanthophyll esterification tended to be higher than those in WT plants. The contents of zeaxanthin + antheraxanthin and β-cryptoxanthin were closely associated with their esterification percentages.

In all *SlXES*-OX plants except #1, contents of lutein, zeaxanthin + antheraxanthin, β-cryptoxanthin, and β-carotene were significantly higher than those in WT plants. Contents of (all-*E*)-neoxanthin, (all-*E*)-violaxanthin, (9′*Z*)-neoxanthin, and (9*Z*)-violaxanthin were significantly lower than or not significantly different from those of WT plants. Esterification percentages of (all-*E*)-neoxanthin, zeaxanthin + antheraxanthin, and β-cryptoxanthin tended to be higher than that in WT plants.

In all *TeXES*-OX plants except #1, contents of (all-*E*)-neoxanthin, (all-*E*)-violaxanthin, (9*Z*)-violaxanthin, lutein, zeaxanthin + antheraxanthin, β-cryptoxanthin, and β-carotene were higher than those in WT plants. The content of (9′*Z*)-neoxanthin was significantly lower than or not significantly different from that of WT plants. Esterification percentages of (all-*E*)-neoxanthin, (all-*E*)-violaxanthin, lutein, zeaxanthin/ antheraxanthin, and β-cryptoxanthin in all plants except for #1 were higher than those in WT plants. Contents of (all-*E*)-neoxanthin, (all-*E*)-violaxanthin, lutein, zeaxanthin + antheraxanthin, and β-cryptoxanthin were closely associated with their esterification percentage.

### Expression analysis of transgenes and endogenous carotenoid metabolic genes

**Figure 4:**
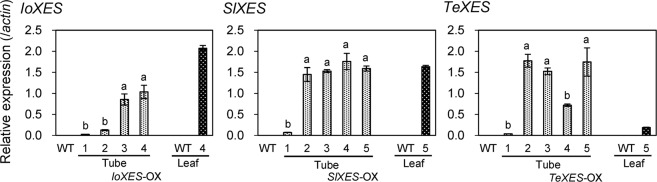
Transgene expression in corolla tubes and leaves of *XES*-OX and WT plants. Data obtained from leaves of *IoXES-*OX #4, *SlXES*-OX #5, and *IoXES*-OX #5 were shown as representatives of each line. RT-qPCR analyses were performed in biological triplicate. Means ± SE are shown. The same letter within a gene indicates no significant difference by Tukey’s test (*P* < 0.05).

The expression levels of transgenes in the corolla tubes and leaves of *XES*-OX lines and WT plants were analyzed by quantitative real-time PCR (RT-qPCR) using primers specific to each *XES* (Fig. 4). No expression of *IoXES*, *SlXES*, or *TeXES* was detected in WT plants. Transgene expression was detected in all *XES*-OX plants. *IoXES*-OX #1, *SlXES*-OX #1, and *TeXES*-OX #1, whose carotenoid contents in tubes were lower than those of WT plants (Fig. 1C), consistently showed lower transgene expression than the other transformants (Fig. 4).

We tested the relationship among the expression of each transgene, esterification percentage, and total carotenoid content in tubes by Pearson’s correlation analysis. There was a very strong positive correlation between *IoXES* expression and esterification percentages (*r* = 0.965, *p* = 0.008; WT was included in all analyses). In contrast, there was a moderate negative correlation between *IoXES* expression and total carotenoid content (*r* = −0.496, *p* = 0.395). The result showed that the *IoXES* expression in *IoXES-OX* plants was closely associated with esterification percentages but not with carotenoid content. There was a very strong positive correlation between *SlXES* expression and esterification percentages (*r* = 0.864, *p* = 0.027), *SlXES* expression and total carotenoid content (*r* = 0.956, *p* = 0.003), *TeXES* expression and esterification percentages (*r* = 0.839, *p* = 0.037), and *TeXES* expression and total carotenoid content (*r* = 0.816, *p* = 0.048). The esterification percentage and total carotenoid content in *SlXES*-OX and *TeXES*-OX plants were significantly associated with transgene expression.

**Figure 5:**
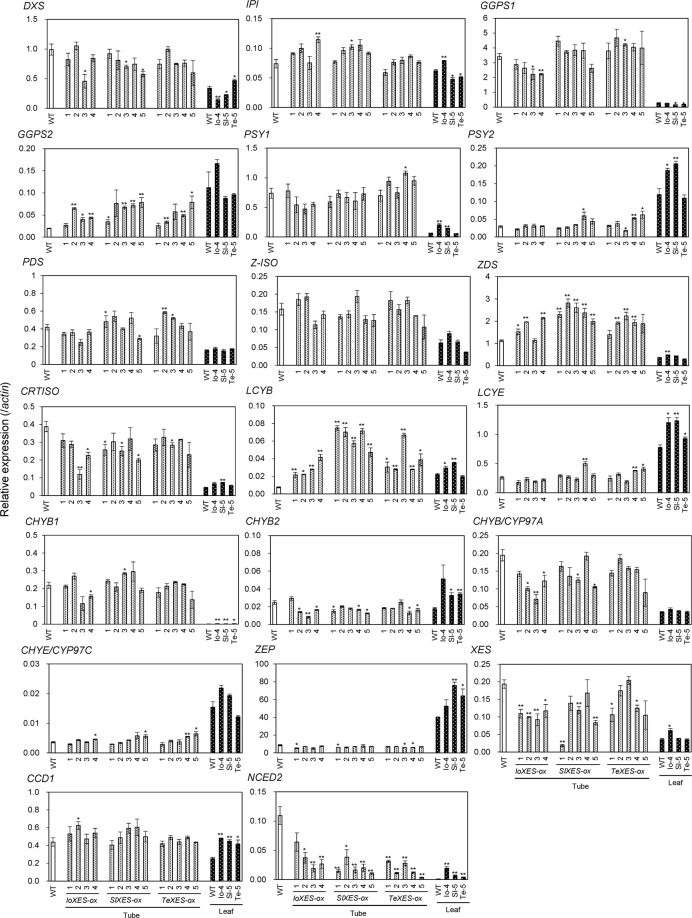
Expression analysis of endogenous carotenoid metabolic genes in corolla tubes and leaves of *XES*-OX and WT plants. Data obtained from leaves of *IoXES*-OX #4, *SlXES*-OX #5, and *IoXES*-OX #5 were shown as representatives of each line. RT-qPCR analyses were performed in biological triplicate. Means ± SE are shown. *P < 0.05, **P < 0.01 versus WT (Student’s *t*-test).

**Figure 6:**
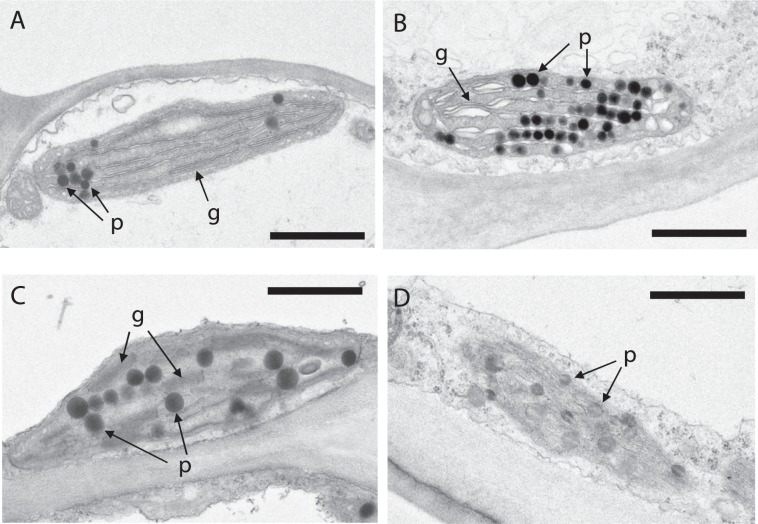
Comparison of plastid ultrastructure in corolla tubes of a WT plant and *TeXES*-OX #5. Ultra-thin sections of tubes were observed by transmission electron microscopy. Plastids from (**A**,**C**) mesophyll cells and (**B**,**D**) epidermal cells from (**A**,**B**) WT and (**C**,**D**) *TeXES*-OX #5 plants are shown. Bars = 1 μm. p, plastoglobule; g, grana.

We then analyzed the expression levels of 20 endogenous carotenoid metabolic genes in the tubes and leaves of *XES*-OX and WT plants (Figs. 5 and 6). Among the genes that encode carotenoid biosynthetic enzymes, *geranylgeranyl diphosphate synthase* (*GGPS*) 2, *ζ-carotene desaturase (ZDS)*, and *lycopene β-ring cyclase (LCYB)* had significantly higher expression in the tubes of almost all (*GGPS2* and *ZDS*) or all (*LCYB*) *XES*-OX plants than WT plants. Expression of *β-ring hydroxylase* (*CHYB*) *2* and petunia endogenous *XES* (*PhXES*) tended to be lower in *XES*-OX plants than in WT plants. Among the carotenoid catabolic genes, we analyzed expression of *carotenoid cleavage dioxygenase* (*CCD*) 1 and *9-*cis*-epoxy carotenoid dioxygenase* (*NCED*) 2, but not *CCD4a* or *CCD4b*, because we have previously clarified that expression of *CCD4a* is not detected at all and expression of *CCD4b* is not associated with carotenoid accumulation in corollas of ‘California Girl’, the petunia cultivar used in this study (Kishimoto *et al*., 2018). *NCED2* expression was lower in all *XES*-OX plants than in WT plants; this trend was significant for all plants except *IoXES*-OX #1. *NCED2* expression tended to be inversely proportional to total carotenoid content. *CCD1* expression showed no significant difference between *XES*-OX and WT plants, except for the significantly higher expression in *IoXES*-OX #2.

### Plastid ultrastructure in the corollas of WT and *XES*-OX plants

By transmission electron microscopy, we examined the plastid ultrastructure in epidermal and mesophyll cells in the corolla tubes of WT plants and *TeXES*-OX #5, which had the highest carotenoid content in both tubes and limbs of all the transgenic plants used in this study (Fig. 1). In WT plants, plastids in mesophyll and epidermal cells had several plastoglobules (globular-type carotenoid-lipoprotein–sequestering substructures) with high electron density inside the granules and a thylakoid membrane with a few grana stacks (Fig. 6A,B). In the plastids in *TeXES*-OX #5 mesophyll cells, the thylakoid membrane was less clear than those in WT mesophyll cells (Fig. 6C). In the plastids in *TeXES*-OX #5 epidermal cells, plastoglobules had lower electron density inside the granules and the thylakoid membrane was hardly visible (Fig. 6D).

## Discussion

We previously reported that *trans*-xanthophylls in petunia corollas have lower esterification percentages than *cis*-xanthophylls, which suggests that PhXES has low substrate specificity for *trans*-xanthophylls^26^. Here, we introduced *XES* genes from *I. obscura*, tomato, and marigold to increase the proportion of esterified xanthophylls in petunia corollas. Petal of these plants accumulate large amounts of *trans*-xanthophylls with various compositions^5,15,17^, which suggest that their XESs have high esterification activity in respect of *trans*-xanthophylls. We previously compared the amino acid sequences of XES in tomato and petunia and found that there are differences in the sequence in the presumed important domains for enzyme activity^26^. We assume that those differences affect the substrate specificity and esterification activity of SlXES and PhXES. We hypothesized that the difference in composition of esterified xanthophylls in *I. obscura*, tomato, and marigold is caused by differences in the substrate specificity of their respective XESs and that by introducing *XES* genes from these species into petunia, we could potentially esterify different types of *trans*-xanthophylls. We showed that the expression of introduced *XES* genes from *I. obscura*, tomato, and marigold affected the proportions of esterified xanthophylls in the tubes and limbs of *XES*-OX corollas (Figs. 1C and 4).

There was a strong positive correlation between esterification percentage and total carotenoid content in *TeXES*-OX and *SlXES*-OX plants (Fig. S3). In *IoXES*-OX plants, esterification percentages tended to be higher than in the *SlXES*-OX and *TeXES*-OX plants; however, the correlation between the esterification percentage and carotenoid content was weaker than that in *TeXES*-OX and *SlXES*-OX plants. In addition, some *IoXES*-OX plants had lower carotenoid content than WT plants despite high esterification percentages. It is necessary to investigate why *IoXES*-OX plants had lower efficiency for xanthophyll ester accumulation than *SlXES*-OX and *TeXES*-OX plants.

There was a strong positive correlation between the expression level of all the transgenes and esterification percentages, and the heterologous expression of *XES* caused an increase in the esterification percentage. However, we considered that xanthophylls in *XES*-OX plants were not efficiently esterified. We used the 35 S promoter to drive the constitutive expression of transgenes and the expression level of the transgenes in tubes was nearly 10 times greater than that of the endogenous *XES* gene, but the maximum esterification percentage was only 66.5% in the tube of *IoXES*-OX #4. If the esterification percentage is increased up to 100% as in marigold, a bright yellow corolla with a total carotenoid content of approximately 200 µg·gFW^−1^ is expected, according to the regression equation obtained in this study (Fig. S3). However, as it does not reach such a high percentage, there are likely to be other factors that further increase the esterification percentage and future research is needed to investigate the other factors.

Introduced *XES* genes were expressed in the leaves of transformants, but xanthophylls contained in the leaves were not esterified at all (Fig. 1C). Ytterberg *et al*. performed proteome analysis in plastoglobules of red pepper and *Arabidopsis* and detected the amino acid sequence of an XES ortholog (homolog of Accession no. At1g54570) and other carotenogenic enzymes^27^. It is presumed that XESs catalyze esterification reactions in hydrophobic conditions inside plastoglobules, in the same way as other carotenoid biosynthetic enzymes in non-green tissues, and chloroplast structures in leaves are inadequate for XES activity.

Petals of the yellow-flowered variety of *I. obscura* predominantly accumulate as (all-*E*)-β-cryptoxanthin, most of which is present in the esterified form^5^. In the petals of marigold, nearly 100% of carotenoids are esterified (all-*E*)-lutein^15^. Reflecting the carotenoid composition in petals of the plant from which the *XES* transgene originated, esterification percentages and accumulation levels of β-cryptoxanthin and lutein were elevated in *IoXES*- and *TeXES*-OX plants, respectively. In the petals of tomato, 85% of xanthophylls are in the esterified form; (all-*E*)-neoxanthin and (all-*E*)-violaxanthin are the main components^17^. However, the esterification percentage of (all-*E*)-neoxanthin and (all-*E*)-violaxanthin did not increase substantially in *SlXES*-OX plants compared with WT. Furthermore, esterified zeaxanthin + antheraxanthin was significantly and dramatically increased, even though zeaxanthin and antheraxanthin are detected in trace amounts in tomato petals. We consider it likely that one factor that prevented the accumulation of esterified neoxanthin and violaxanthin in the *SlXES*-OX plants was the low biosynthesis of these xanthophylls. Our expression analysis showed that the transcript level of the *zeaxanthin epoxidase* (*ZEP*) was low in the tubes of *XES*-OX and WT plants. Low ZEP activity might have prevented the conversion of zeaxanthin to violaxanthin, which resulted in increased accumulation of zeaxanthin. The effect of low *ZEP* expression on carotenoid accumulation is also discussed below. In the *TeXES*-OX plants, unlike the *IoXES*-OX and *SlXES*-OX plants, a slight increase in violaxanthin and neoxanthin in both *trans*- and *cis*-forms was observed. This may be because TeXES mainly targets lutein, an α-carotene derivative. As IoXES and SlXES mainly target β-carotene derivatives, biosynthetic intermediates, such as β-cryptoxanthin and zeaxanthin, may be converted to the ester form before reaching neoxanthin and violaxanthin. In contrast, it is likely that TeXES has a lower effect on β-carotene derivatives than the other two XESs, and neoxanthin and violaxanthin are increased regardless of low ZEP activity.

We presume that esterified xanthophylls may not continue in the steps of carotenoid biosynthesis because of their sequestration into plastoglobules or the substrate specificity of carotenoid biosynthetic enzymes, such as by hydroxylation and epoxidation enzymes. This means that xanthophylls preferentially esterified by XES may be dominantly accumulated. Here, we showed that *IoXES* plants preferably esterified β-cryptoxanthin and enhanced its accumulation in petunia corollas, which originally accumulate only a trace amount. β-Cryptoxanthin, which is formed by the hydroxylation of one of the β-rings of β-carotene by CHYB, has attracted much attention as a functional and nutritional component^28–30^. When humans ingest esterified β-cryptoxanthin, an ester bond is cleaved during digestion. The concentration of free β-cryptoxanthin in the blood after ingestion increases regardless of whether it was an ester form or free form. Therefore, the presence or absence of an ester bond does not affect the function of nutrient components^31^. However, only a few plants are known to accumulate β-cryptoxanthin^32^, presumably because most plants have XES orthologs with no or low esterification activity for β-cryptoxanthin. In plants without this activity, free-form β-cryptoxanthin is expected to be continuously hydroxylated to form zeaxanthin. In contrast, in plants with high levels of this activity, such as *IoXES*-OX plants and *I. obscura*, β-cryptoxanthin can be esterified, which prevents it from being converted to zeaxanthin. Therefore, we propose that *IoXES* may be a powerful tool for enhancing the accumulation of β-cryptoxanthin in flowers and fruits containing xanthophylls.

In our comparison of the ultrastructure of plastids (the carotenoid storage organelles) between *TeXES*-OX #5 and WT plants (Fig. 6), we observed that the plastoglobules in both epidermal and mesophyll cells of *TeXES*-OX #5 had lower electron densities than those in WT plants. Ariizumi *et al*. previously reported the same tendency: plastids in petals of WT tomato plants are filled with numerous plastoglobules with low electron density, whereas those in *pyp1* plants, which lack XES activity, contain small and high-electron-density plastoglobules^17^. We assume that, in *XES* plants, an increase in the accumulation of esterified xanthophylls into plastoglobules causes a decrease in their electron density.

**Figure 7:**
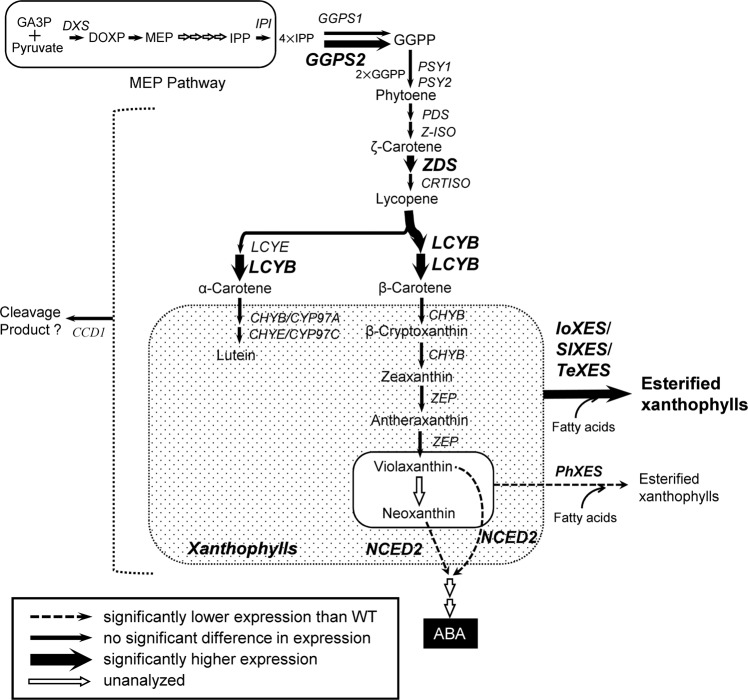
Typical putative carotenoid biosynthetic pathway in corollas of *IoXES*-OX, *SlXES*-OX, and *TeXES*-OX plants. GA3P, glyceraldehyde 3-phosphate; DXS, 1-deoxy-d-xylulose 5-phosphate synthase; DOXP, 1-deoxy-d-xylulose 5-phosphate; MEP, 2-C-methyl-d-erythritol-2,4-cyclodisphosphate; IPP, isopentenyl diphosphate; IPI, IPP isomerase; GGPP, geranylgeranyl diphosphate; GGPS, GGPP synthase; PSY, phytoene synthase; PDS, phytoene desaturase; Z-ISO, 15-*cis*-ζ-carotene isomerase; ZDS, ζ-carotene desaturase; CRTISO, carotenoid isomerase; LCYB, lycopene β-ring cyclase; LCYE, lycopene ε-ring cyclase; CHYB, β-ring hydroxylase; CHYE, ε-ring hydroxylase; CHYB/CYP97A, cytochrome P450-type β-ring hydroxylase; CHYE/CYP97C, cytochrome P450-type ε-ring hydroxylase; ZEP, zeaxanthin epoxidase; NCED2, 9-*cis*-epoxy carotenoid dioxygenase 2; ABA, abscisic acid; CCD1, carotenoid cleavage dioxygenase 1; PhXES, *Petunia hybrida* xanthophyll esterase; IoXES, *Ipomoea obscura* XES; SlXES, *Solanum lycopersicum* XES; TeXES, *Tagetes erecta* XES. Expression of genes in bold was enhanced when *XES* genes were exogenously introduced.

There is increasing evidence that feedback and feed-forward mechanisms play important roles in the regulation of the carotenoid biosynthetic pathway^33,34^. Recently, in tomato, the interaction between various substrates and enzyme activities has become apparent by introducing heterologous carotenoid biosynthetic genes into carotenoid deficient mutants, e.g., *tangerine* (a loss-of-function mutant of *carotenoid isomerase*) and *old gold crimson* (a loss-of-function mutant of fruit-specific *lycopene β-cyclase*)^35,36^. For example, the introduction of the *bacterial desaturase*/*isomerase gene* (*CRTI*) into tomato deficient mutants reveals that the decrease in phytoene, which is a substrate for CRTI, causes negative feedback regulation and results in the decreased expression of *PSY*. Simultaneously, it became clear that the increase in *trans*-lycopene, a CRTI product, also causes feed-forward regulation that increases the expression of *lycopene cyclases*^35^. It is speculated that the reason cultivated carrots accumulate a higher amount of α-carotene than wild carrots is due to the positive feedback regulation of *PSY* expression caused by the loss-of-function mutation of *cytochrome P450-type β-ring hydroxylase* (*CYP97A3*)^37^. Among the endogenous carotenoid metabolic genes tested in this study (Fig. 5), the levels of the transcripts of the carotenoid biosynthetic genes (*GGPS2*, *ZDS*, and *LCYB*) were significantly higher in most of the *XES*-OX plants that had higher carotenoid content than those in WT plants. The putative carotenoid biosynthetic pathway in the corollas of *XES*-OX plants is shown in Fig. 7. We previously showed that the expression of *PSY1* and *lycopene ε-ring cyclase* in petunia cultivars is significantly higher in pale yellow corollas than in white corollas^13^. We also showed that the higher carotenoid content in tubes than that in limbs in both white and pale-yellow corollas is associated with higher expression of *GGPS1*, *PSY1*, *carotenoid isomerase*, *lycopene ε-ring cyclase*, and *cytochrome P450-type β-ring hydroxylase*. The genes that showed higher expression in the *XES*-OX plants than that in the WT plants in this study are different from those that showed higher expression in tubes than in limbs in the previous study^26^. Therefore, we presume that the mechanism by which carotenoid accumulation is controlled in corollas differs between *XES*-OX plants and petunia cultivars. The increased expression of genes in *XES*-OX plants was likely to be induced by feedback from the accumulation of esterified xanthophylls or overall carotenoids. In deep-yellow-flowered calibrachoa, the content of β-carotene is higher than that in WT petunia^26^, asas we observed in *SlXES*-OX and *TeXES*-OX plants. The expression levels of *isopentenyl diphosphate isomerase*, *PSY1*, *LCYB*, *cytochrome P450-type ε-ring hydroxylase*, *ZEP*, and *XES* are higher in calibrachoa than those in petunia. A higher expression level of *LCYB* was observed in both calibrachoa^26^ and *XES*-OX plants (Fig. 5) than that in WT petunia plants, which suggested that LCYB is a key enzyme in the synthesis of β,β-carotenoids (β-carotene and its derivatives) in the corollas of calibrachoa and petunia. In the *SlXES*-OX plants, the consistently higher contents of β,β-carotenoids, such as β-carotene and zeaxanthin + antheraxanthin, might be due to the higher expression of *LCYB* in these plants than that in *IoXES*-OX and *TeXES*-OX plants (Figs. 3 and 5). Here, we propose that both the increased percentage of xanthophyll esterification and the enhanced carotenoid biosynthetic gene expression cause an increase in the carotenoid accumulation in *XES*-OX plants. *XES*-OX plants did not reach the carotenoid level observed in calibrachoa corollas, perhaps because the expression of *ZEP* was lower in both *XES*-OX and WT plants than that in calibrachoa (Figs. 1C, 5 and 6)^26^. PhXES and SlXES are expected to have a higher esterification activity in regard to neoxanthin and violaxanthin than that of the other xanthophylls because of the high esterification percentage of xanthophylls in petunia^26^ and tomato^17^. However, *SlXES*-OX plants cannot utilize the ability to esterify neoxanthin and violaxanthin due to the low accumulation of these two components. If *ZEP* expression was to be increased in the corollas of *XES*-OX and WT plants, we consider it likely that the biosynthesis of neoxanthin and violaxanthin would be elevated and that the esterified forms of neoxanthin and violaxanthin would increase. This increase in xanthophyll esters would further promote biosynthetic gene expression and the development of plastoglobules in chromoplasts, which would result in a further increase in total carotenoid accumulation.

Our expression analysis of carotenoid catabolic genes showed that xanthophyll esterification was also associated with *NCED2* expression in *XES*-OX plants. *NCED2* had a significantly lower expression in *XES*-OX plants than that in the WT plants and total carotenoid content of *XES*-OX plants tended to be inversely proportional to *NCED2* expression levels (Figs. 1C and 5). NCED is localized in chloroplast thylakoid membranes and cleaves (9*Z*)-violaxanthin and (9′*Z*)-neoxanthin to xanthoxin, which is a precursor of abscisic acid^38,39^. However, the neoxanthin and violaxanthin content in *IoXES*-OX and *SlXES*-OX plants was not higher than those in WT plants (Fig. 3). It is possible that NCED2 is unable to cleave violaxanthin and neoxanthin in the plastoglobules of chromoplast and that the expression level of *NCED2* is independent of the amount of violaxanthin and neoxanthin in the corollas.

## Conclusion

The main effects that introducing *XES* genes from different species had on petunia corollas were promotion of xanthophyll esterification and increase in *LCYB* expression, possibly caused by feedback regulation. These multiple effects likely caused the elevated carotenoid accumulation in corollas of *XES*-OX plants. Only a few attempts at manipulation of carotenoid accumulation in petals by genetic engineering have been reported. Watanabe *et al*. introduced a set of carotenoid biosynthetic genes into white-flowered *Ipomoea nil* with the intention of producing yellow flowers^40^, since the expression of many carotenogenic genes is suppressed in its petals^5^, but although the carotenoid content in the petals of the transformants increased to ~1 μg/g FW, it was insufficient to make the flowers visually yellow. These results indicate that carotenoid biosynthetic activity has limited effect on carotenoid accumulation in petals. A number of genes other than carotenogenic genes influence carotenoid accumulation, making metabolic engineering of carotenoids difficult. Here we showed that introducing a single gene, *XES*, could clearly change the petal color of petunia (Fig. 1A). We also showed that the substrate specificity of introduced *XES*s had a marked effect on the carotenoid composition in the transformants. We suggest that substrate specificity is a reason for the diverse carotenoid profiles among different plant species. To control the accumulation of specific carotenoids, further study of the relationship between XES activity and carotenoid composition is needed.

## Methods

### Plant materials

Transgenic plants were produced from the pale-yellow-flowered petunia cultivar ‘California Girl’. Seedlings were grown on Murashige-Skoog agar medium with half-strength minerals at 25 °C under a 16-h light/8-h dark photoperiod with fluorescent light (120 μmol m^−2^ s^−1^). The fully-grown seedlings were applied to *Agrobacterium*-mediated transformation or transferred to soil and grown in a greenhouse at the Institute of Vegetable and Floriculture Science, NARO (Tsukuba, Japan). The second to fifth flowers, in order of flowering time, in each plant were sampled from 9 to 11 a.m. on the flowering day. Three corollas were harvested and divided into limbs and tubes. They were further divided vertically; one used for carotenoid analysis and the other for gene expression analysis. Mature leaves were sampled and stored at −80 °C.

For isolation of genes, a bright-yellow-flowered variety of *I. obscura* ‘Keniaki’, tomato ‘Mini Carol’, and orange-flowered marigold ‘Orange Lady’ were grown in greenhouses. Petals from fully opened flowers were sampled and stored at −80 °C until use.

### Cloning of full-length *XES* genes

We isolated full-length cDNA sequences of *XES*, an ortholog of tomato *PYP1*, from *I. obscura* and marigold by 5′ RACE and 3′ RACE as described by Kishimoto *et al*.^13^, using the primer sequences shown in Table S1. We isolated the full-length *SlXES* cDNA sequence by conducting RT-PCR using the conditions described by Kishimoto *et al*.^26^ and 5′- and 3′-end primers designed from the published sequence (acc. no. XM_004230093; Table S1). The full-length cDNA sequences of *IoXES* (LC335777) and *TeXES* (LC335776) are available in the DDBJ/EMBL/GenBank nucleotide sequence database.

The deduced amino acid sequences of the *XES* genes identified in this study were compared with those in the database by using the neighbor-joining method and bootstrap analysis (1000 replicates) in PHYLIP software^41^ and were mapped in Treeview v. 1.6.6 software^42^.

### Vector construction and *Agrobacterium*-mediated transformation

A binary vector, pRI201-AN (TaKaRa, Shiga, Japan), containing the CaMV 35 S promoter was used for *Agrobacterium*-mediated transformation. The vector was digested with *Sac*I and *EcoR*I, and the heat shock protein (HSP) terminator was replaced with the HSPT878 terminator^43^. Full-length fragments of *IoXES*, *SlXES*, and *TeXES* cDNAs were directly linked downstream of the 5′-untranslated region of a *Nicotiana tabacum alcohol dehydrogenase* (*NtADH-*5′UTR) fragment, and an *Xba*I site at the 5′ end and a *Sac*I site at 3′ end were added by the overlap extension PCR method^44^. The resultant *NtADH*-5′UTR–*XES* fragments were inserted into the *Xba*I/*Sac*I sites of the modified pRI201-AN (Fig. S2).

*Agrobacterium*-mediated transformation was performed as described by Jorgensen *et al*.^45^, using *Agrobacterium* strain EHA105 carrying the above constructs. Transformed petunia plants were selected for kanamycin resistance, and resistant shoots were then transferred to soil and were used for analysis (T_0_ generation).

### RT-qPCR analysis

Isolation of total RNAs and reverse-transcription RT-qPCR analysis in corolla tubes and limbs were performed as described by Kishimoto *et al*.^13^ using reported primers for RT-qPCR of endogenous carotenoid metabolic genes^13,26^. Primers specific to *PhXES*, *IoXES*, *SlXES*, and *TeXES* were designed in Oligo software (Molecular Biology Insights, CO, USA) by comparing the full-length cDNAs (Table S2); we confirmed that the primers could not amplify endogenous *PhXES* transcripts. All analyses were performed in biological triplicate. Statistically significant differences between petunia transformants and WT plants were determined by two-tailed Student’s *t*-test and Tukey’s test at the 5% level.

### HPLC analysis of carotenoids

HPLC analysis conditions and peak identification were as described by Kishimoto *et al*.^26^. Measurements were performed in biological triplicate. Statistically significant differences between petunia transformants and WT plants were determined by unpaired two-tailed Student’s *t*-test at the 5% level. The relationship between the expression levels of exogenous *XES* genes, percentages of xanthophyll esterification, and total carotenoid contents were tested by Pearson’s correlation analysis.

### Transmission electron microscopy

Tubes were cut into small pieces (~1 mm^3^), fixed, dehydrated, and embedded in Quetol 651 (Nisshin EM Co., Tokyo, Japan) as described by Ohmiya *et al*.^46^. Ultrathin sections were cut with a diamond knife on an ultramicrotome (Ultracut UCT, Leica Microsystems, Wetzlar, Germany). Sections were picked up on copper grids, stained with uranyl acetate and lead citrate, and observed under a transmission electron microscope (JEM-1200EX; JEOL Ltd., Tokyo, Japan) at an acceleration voltage of 80 kV. Digital images were taken with a CCD camera (Veleta; Olympus Soft Imaging Solutions GmbH, Münster, Germany).

### Accession codes

The nucleotide sequence reported in this paper has been submitted to DDBJ/EMBL/GenBank under accession numbers LC335777 (*Ipomoea obscura* XES) and LC335776 (marigold XES).

## Supplementary information

Supplementary information.
