# Supplementary material for: Heterologous expression of xanthophyll esterase genes affects carotenoid accumulation in petunia corollas
Source: Sci Rep. 2020 Jan 28;10:1299. doi: 10.1038/s41598-020-58313-y (PMC6987204; doi:10.1038/s41598-020-58313-y)
Supplement: Supplementary file 1 — Supplementary information. [file 41598_2020_58313_MOESM1_ESM.pdf]

Supplementary Informations

Title: Heterologous expression of xanthophyll esterase genes affects carotenoid accumulation in petunia corollas.

Authors: Sanae Kishimoto, Chihiro Oda-Yamamizo and Akemi Ohmiya

Supplementary Table S1. Primers used for cloning of *XES* s.

Gene	I. D.	Sequence
IoXES	GSP1	CCACCGGGATAGAGGAGCACATGA
	GSP2	CCTCCTTGAACGGCGATATGTGCT
	5' end-1F (3' RACE)	TTGGGAAGCATTGATTTTAGAGCCC
	5' end-2F (3' RACE)	ATGGCTTTGATTATGCAGAGTTTTAAATTA
TeXES	GSP1	GCCGGATGTGCTATACCACGAACGA
	GSP2	AAATAAGTGGCGCGAGTTCTAACCC
	5' end-1F (3' RACE)	CACATTCACCCAATTCTTGTAAAACAGC
	5' end-2F (3' RACE)	ATGTTTGTAAAAGCTACAGCTTCAATTG
SlXES	F	ATGGCTTCTCTTCTGCATAATTTC
	R	TCATCTAAAAATCAAATGTTGGGACA

F, forward; R, reverse

Supplementary Table S2. Primers for RT-qPCR of introduced *XES* s.

Target gene	Direction	Sequence
IoXES	Forward	TGGAGTAGCACACCCAACATGT
	Reverse	AGGTCGGTCTGGCCACATT
SlXES	Forward	CATCCATCGTTGTTTACTCAGCTGG
	Reverse	AGCCATTCTGATGAATTCTGGTTGG
TeXES	Forward	CCGGCACTTTTCACAAGGAGTTC
	Reverse	TTCTGGTTGATCAGGCCAAAACAA

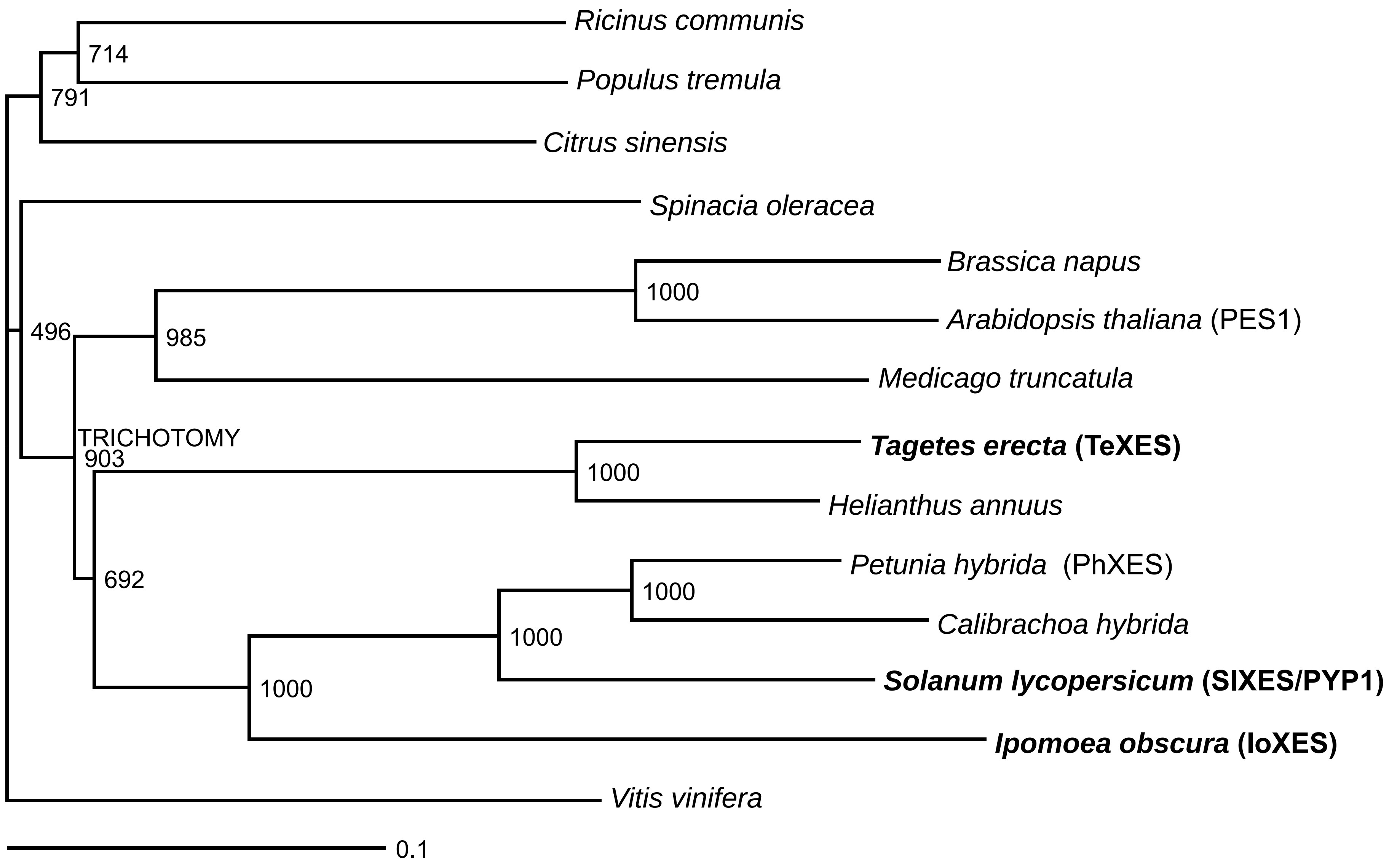

Supplementary Figure S1. Alignment of deduced amino acid sequences of orthologs of *Solanum lycopersicum* SlXES/PYP1 (XM_004230093) in various plant species: *Arabidopsis thaliana* PES1 (NM_104335); *Brassica napus* (XM_013894821); *Calibrachoa hybrida* (LC335779); *Citrus sinensis* (XM_006471720); *Helianthus annuus* (XM_022154563); *Ipomoea obscura* IoXES (LC335777); *Medicago truncatula* (XM_003626323); *Petunia hybrida* PhXES (LC335778); *Populus tremula* (XM_006382665); *Ricinus communis* (XM_002512225); *Tagetes erecta* TeXES (LC335776); *Vitis vinifera* (XM_002271416). Numbers at branch points indicate bootstrap values (1000 replicates). XESs examined in this study are indicated in bold.

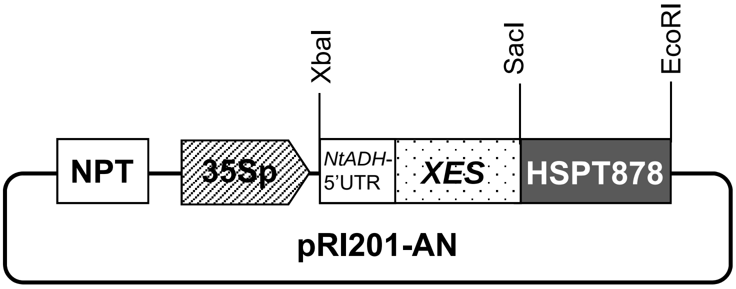

Supplementary Figure S2. *XES*-OX construction used in this study.

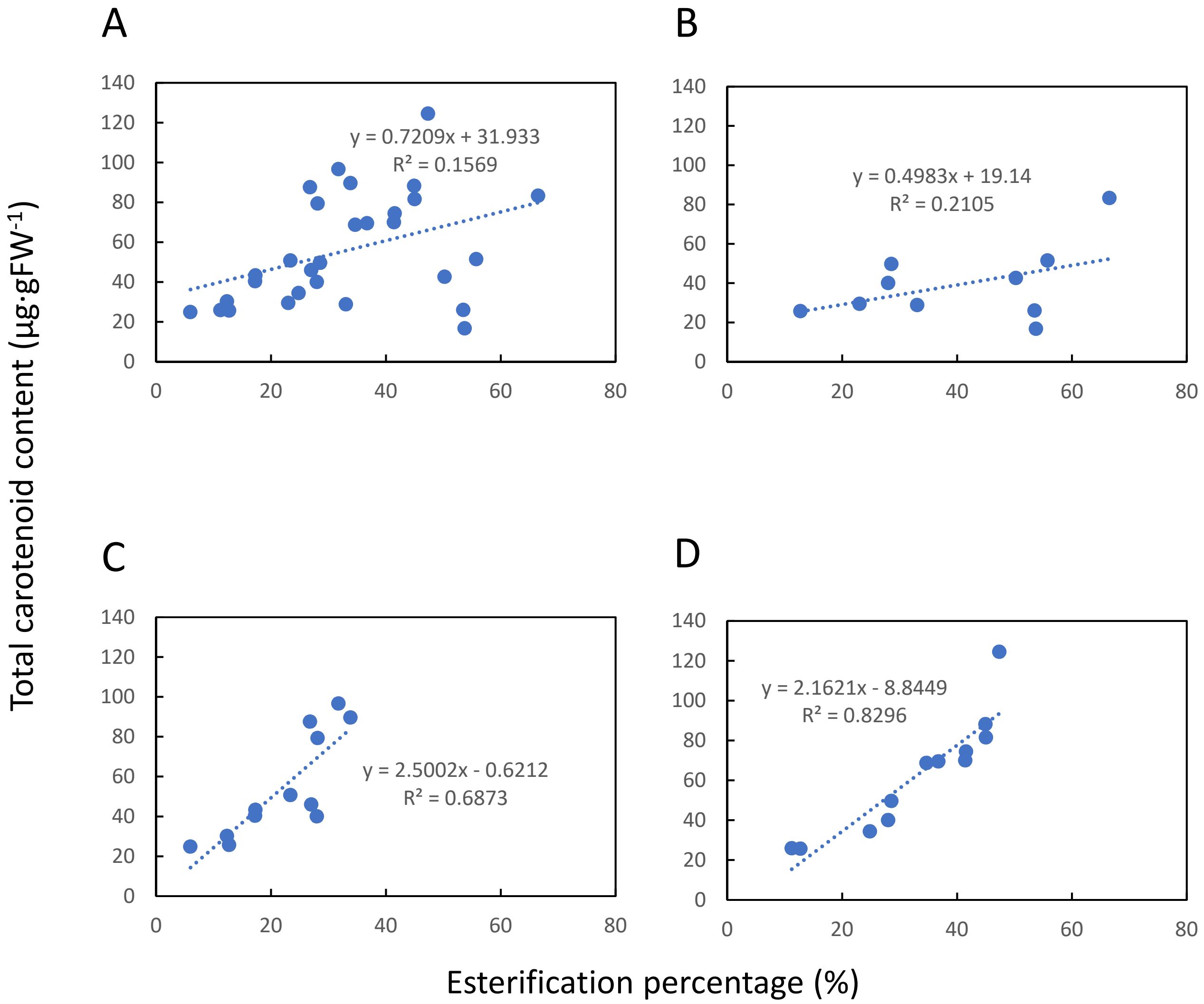

Supplementary Figure S3. The relationship between total carotenoid content and percentage of xanthophyll esterification in limbs and tubes of *XES*-OX and WT plants. The correlation was tested by Pearson's correlation analysis. Limbs and tubes of (A) all *XES*-OX and WT plants, (B) *IoXES*-OX and WT plants, (C) *SlXES*-OX and WT plants, and (D) *TeXES-OX* and WT plants.
